# Supplementary material for: The histone chaperone SPT2 regulates chromatin structure and function in Metazoa
Source: Nat Struct Mol Biol. Author manuscript; Available in PMC 2024 Mar 18. (PMC7615752; doi:10.1038/s41594-023-01204-3)
Supplement: Supplementary Figure Legend [file EMS194104-supplement-Supplementary_Figure_Legend.pdf]

nature structural & molecular biology

Article https://doi.org/10.1038/s41594-023-01204-3

The histone chaperone SPT2 regulates chromatin structure and function in Metazoa

In the format provided by the authors and unedited

Supplementary Figure Legend

Definition of species abbreviations, relevant to Extended Data Figure 1a

SPT2_HUMAN, *Homo sapiens*; SPT2_MOUSE, *Mus musculus*; SPT2_CHICK, *Gallus gallus*; SPT2_XENTR, *Xenopus tropicalis*; SPT2_DANRE, D*anio rerio*; A0A6F9DU61_9ASCI, *Phallusia mammillata*; A0A7M7GFY6_STRPU, *Strongylocentrotus purpuratus*; SPT2_DROME, *Drosophila melanogaster*; A0A158NW08_ATTCE, *Atta cephalotes*; A0A0A9XSF3_LYGHE, *Lygus hesperus*; A0A7E5X4U9_TRINI, *Trichoplusia ni*; A0A5B7DAY1_PORTR, *Portunus trituberculatus*; A0A6P9FP65_IXOSC, *Ixodes scapularis*; A0A3S3P368_9ACAR, *Dinothrombium tinctorium*; A0A6P6Y5L7_DERPT, *Dermatophagoides pteronyssinus*; A0A6P7SLJ7_OCTVU, *Octopus vulgaris*; A0A1S3JQ94_LINUN, *Lingula unguis*; R7VB03_CAPTE, *Capitella teleta*; Q9GYK8_CAEEL, *Caenorhabditis elegans*; A8X1T8_CAEBR, *Caenorhabditis briggsae*; A0A2P4W7N5_CAERE, *Caenorhabditis remanei*; CAB3406877_CAEBO, *Caenorhabditis bovis*; A0A2A2LZT0_9BILA, *Diploscapter pachys*; A0A7I4YH54_HAECO, *Haemonchus contortus*; A0A2A6CB70_PRIPA, *Pristionchus pacificus*; A0A1I7RM09_BURXY, *Bursaphelenchus xylophilus*; A0A1I7Y028_9BILA, *Steinernema glaseri*; A0A0N4UT22_ENTVE, *Enterobius vermicularis*; A0A1I7V827_LOALO, *Loa loa*; A0A3Q0KDM5_SCHMA, *Schistosoma mansoni*; A0A4E0S0M5_FASHE, *Fasciola hepatica*; A0A3P7LA61_DIBLA, *Dibothriocephalus latus*; T2MBP2_HYDVU, *Hydra vulgaris*; A7RLI9_NEMVE, *Nematostella vectensis*; A0A1X7U7Q7_AMPQE, *Amphimedon queenslandica*.
