# Supplementary material for: The histone chaperone SPT2 regulates chromatin structure and function in Metazoa
Source: Nat Struct Mol Biol. 2024 Jan 18;31(3):523–35. doi: 10.1038/s41594-023-01204-3 (PMC7615752; doi:10.1038/s41594-023-01204-3)

Source Data - Uncropped gels and western blot images relative to Extended Data Fig. 1

Fig. 1c

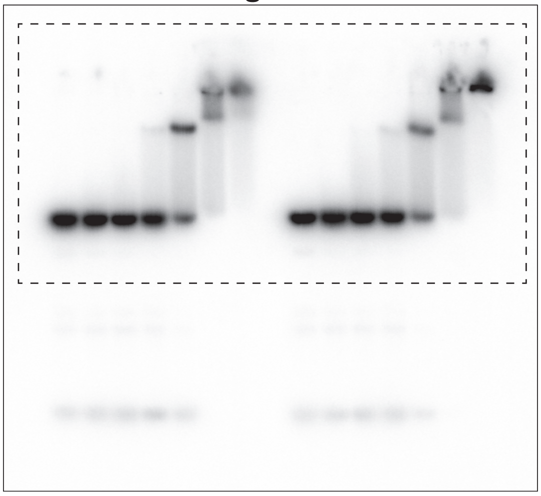

Fig. 1d

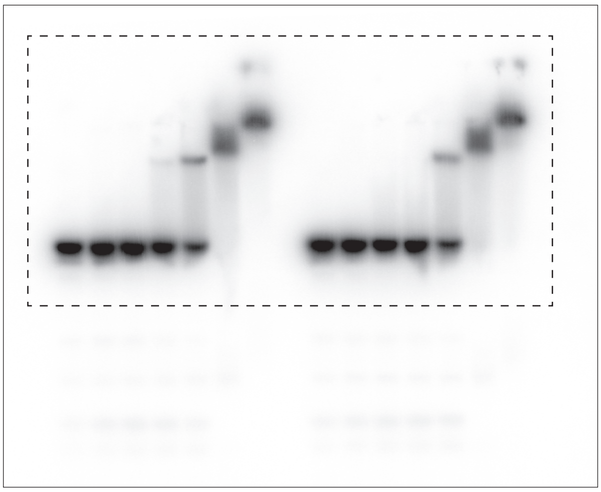

Supplement: Supplementary file 2 [file 41594_2023_1204_MOESM14_ESM.pdf]
