# Supplementary material for: The histone chaperone SPT2 regulates chromatin structure and function in Metazoa
Source: Nat Struct Mol Biol. 2024 Jan 18;31(3):523–35. doi: 10.1038/s41594-023-01204-3 (PMC7615752; doi:10.1038/s41594-023-01204-3)

Source Data - Uncropped gels and western blot images relative to Extended Data Fig. 6

Fig. 6a

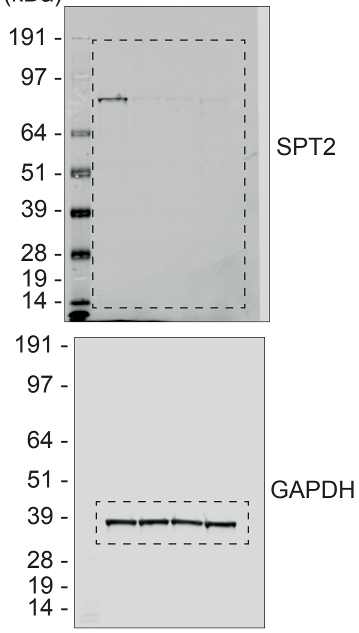

Fig. 6b

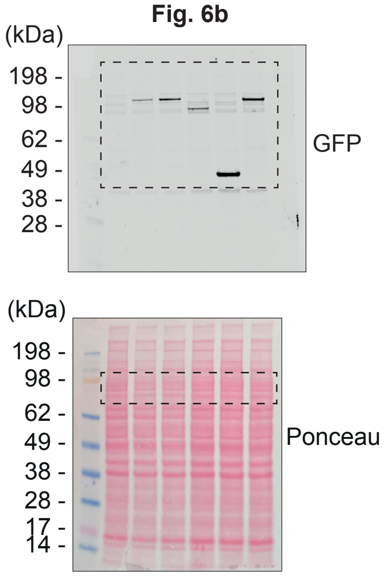

Fig. 6c

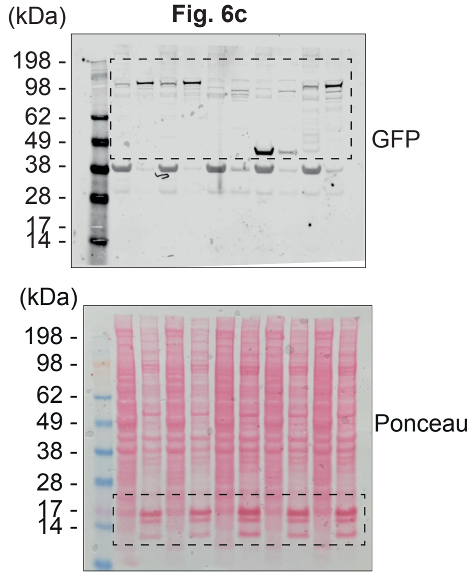

Fig. 6f

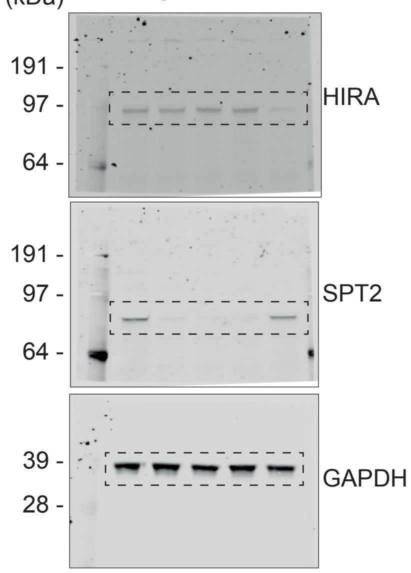

Fig. 6g

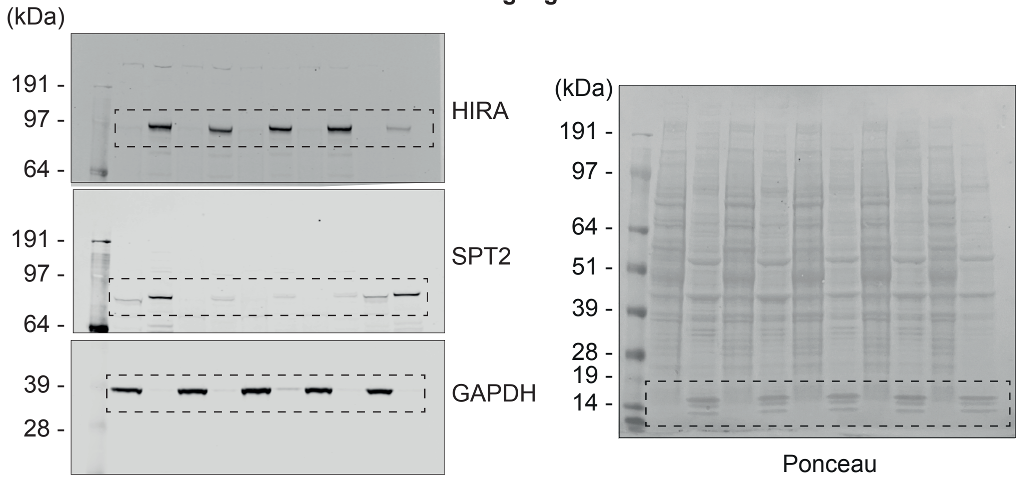

Fig. 6i

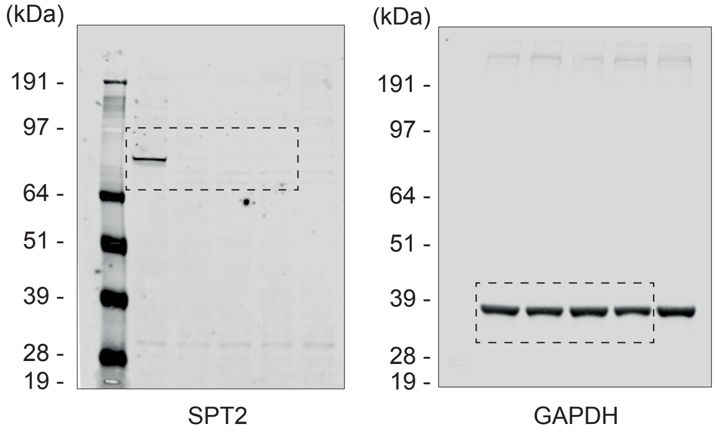

Supplement: Supplementary file 3 [file 41594_2023_1204_MOESM18_ESM.pdf]
