# Supplementary material for: The histone chaperone SPT2 regulates chromatin structure and function in Metazoa
Source: Nat Struct Mol Biol. 2024 Jan 18;31(3):523–35. doi: 10.1038/s41594-023-01204-3 (PMC7615752; doi:10.1038/s41594-023-01204-3)
Supplement: Supplementary file 4 [file 41594_2023_1204_MOESM2_ESM.pdf]

nature portfolio

Corresponding author(s): John Rouse, Giulia Saredi

Last updated by author(s): Nov 27, 2023

Reporting Summary

Nature Portfolio wishes to improve the reproducibility of the work that we publish. This form provides structure for consistency and transparency in reporting. For further information on Nature Portfolio policies, see our Editorial Policies and the Editorial Policy Checklist.

Statistics

For all statistical analyses, confirm that the following items are present in the figure legend, table legend, main text, or Methods section.

n/a	Confirmed	
☐	☒	The exact sample size (*n*) for each experimental group/condition, given as a discrete number and unit of measurement
☐	☒	A statement on whether measurements were taken from distinct samples or whether the same sample was measured repeatedly
☐	☒	The statistical test(s) used AND whether they are one- or two-sided<br>*Only common tests should be described solely by name; describe more complex techniques in the Methods section.*
☒	☐	A description of all covariates tested
☐	☒	A description of any assumptions or corrections, such as tests of normality and adjustment for multiple comparisons
☐	☒	A full description of the statistical parameters including central tendency (e.g. means) or other basic estimates (e.g. regression coefficient) AND variation (e.g. standard deviation) or associated estimates of uncertainty (e.g. confidence intervals)
☐	☒	For null hypothesis testing, the test statistic (e.g. *F*, *t*, *r*) with confidence intervals, effect sizes, degrees of freedom and *P* value noted<br>*Give P values as exact values whenever suitable.*
☒	☐	For Bayesian analysis, information on the choice of priors and Markov chain Monte Carlo settings
☒	☐	For hierarchical and complex designs, identification of the appropriate level for tests and full reporting of outcomes
☒	☐	Estimates of effect sizes (e.g. Cohen's *d*, Pearson's *r*), indicating how they were calculated

Our web collection on statistics for biologists contains articles on many of the points above.

Software and code

Policy information about availability of computer code

Data collection	Microscopy image acquisition was performed with an Imager M2 (Carl Zeiss) (C. elegans images in Fig. 2g-j); an SP8 confocal fluorescence microscope (Leica) (Fig. 3b,e); a Perkin Elmer Operetta imaging software (Fig. 6b, right); an Olympus ScanR automated microscope (Fig. 6b, e,f; Extended Data Fig. 6j,k); a DMI6000 epifluorescence microscope (Leica) and MetaMorph Software (Extended Data Fig. 6d,e). An Odissey CLx LI-COR for used for western blotting. A Typhoon FLA 9500 phosphorimager was used to image EMSA gels; qPCR was performed using a CFX384 real-time PCR system (Bio-Rad).
Data analysis	Statistic analysis was performed using Prism 9. Western blot processing was performed using LI-COR EmpiriaStudio software (version 2.3.0.154). Data analysis relied on published software as described in the Methods section.

For manuscripts utilizing custom algorithms or software that are central to the research but not yet described in published literature, software must be made available to editors and reviewers. We strongly encourage code deposition in a community repository (e.g. GitHub). See the Nature Portfolio guidelines for submitting code & software for further information.

Data

Policy information about availability of data

All manuscripts must include a data availability statement. This statement should provide the following information, where applicable:

- Accession codes, unique identifiers, or web links for publicly available datasets
- A description of any restrictions on data availability
- For clinical datasets or third party data, please ensure that the statement adheres to our policy

All plasmids and antibodies generated in this study can be requested to the MRC PPU DSTT: https://mrcppureagents.dundee.ac.uk/reagents-from-papers/Rouse-SPT2-paper-1
All NGS datasets have been deposited on GEO with accession number GSE224802.
The previously published crystal structure of SPT2 HBD in complex with H3-H4 was retrieved from PDB under the ID 5BS7.
Other materials generated in this work will be made available upon request.

Human research participants

Policy information about studies involving human research participants and Sex and Gender in Research.

Reporting on sex and gender	No human research participants were used in this study.
Population characteristics	No human research participants were used in this study.
Recruitment	No human research participants were used in this study.
Ethics oversight	No human research participants were used in this study.

Note that full information on the approval of the study protocol must also be provided in the manuscript.

Field-specific reporting

Please select the one below that is the best fit for your research. If you are not sure, read the appropriate sections before making your selection.

☒ Life sciences ☐ Behavioural & social sciences ☐ Ecological, evolutionary & environmental sciences

For a reference copy of the document with all sections, see nature.com/documents/nr-reporting-summary-flat.pdf

Life sciences study design

All studies must disclose on these points even when the disclosure is negative.

Sample size	The sample size used in each experiment was not predetermined or formally justified for statistical power. Samples sizes used in our experiments followed the conventions used in the field.
Data exclusions	Three mRNA-seq samples were removed after quality control as they did not meet the standard to be used for differential gene expression analysis (also described in the Methods section).<br>Three human ATAC-seq samples were removed: Two replicates (SPT2 KO-6 Replicate 2 and SPT2 KO-4 Replicate 1) were excluded from the analysis due to a lower signal to noise ratio compared to other replicates from KO strains. A wild type sample (WT Replicate 3) was also excluded as it showed differences in its accessibility profile compared to the other wild type replicates. These three excluded samples all clustered separately from other wild-type and mutant samples from the same strain both in PCA and in correlation heatmaps (also described in the Methods section).
Replication	The number of replicates for each experiments are indicated in the relevant figure legend.
Randomization	Sample randomization was not applicable in this study; as far as we know, randomization is not something that is applied to the kind of experiments that we present in this paper.
Blinding	With the exception of the experiment in Fig. 5b, no blinding was performed in this study. All our samples were however processed in parallel and the analysis was independent of the genotype or condition used.

Reporting for specific materials, systems and methods

We require information from authors about some types of materials, experimental systems and methods used in many studies. Here, indicate whether each material, system or method listed is relevant to your study. If you are not sure if a list item applies to your research, read the appropriate section before selecting a response.

Materials & experimental systems

n/a	Involved in the study
☐	☒ Antibodies
☐	☒ Eukaryotic cell lines
☒	☐ Palaeontology and archaeology
☐	☒ Animals and other organisms
☒	☐ Clinical data
☒	☐ Dual use research of concern

Methods

n/a	Involved in the study
☐	☒ ChIP-seq
☒	☐ Flow cytometry
☒	☐ MRI-based neuroimaging

Antibodies

Antibodies used	Primary antibodies: Human SPT2 (in-house produced, sheep DA010, 3rd bleed; WB: 1:2000, IF: 1:1000); GAPDH (Cell Signalling, clone 14C10, 2118S; WB: 1:5000); GFP (Abcam, ab290; WB: 1:2000); RPB1 (Cell Signalling, clone D8L4Y, 14958S; WB: 1:2000); HIRA (Active Motif, 39457; WB: 1:1000); His6 (Abcam, ab18184; 1:1000), H3 (Abcam, ab1971; 1:1000).<br>Secondary antibodies: IRDye antibodies (LI-COR) for western blotting, 1:15,000; Alexa Fluor antibodies (ThermoFisher) for immunofluorescence, 1:1000.
Validation	The human SPT2 antibody was validated by siRNA-mediated depletion of SPT2 (Extended Data Fig. 6a).<br>The HIRA 39457 antibody was validated by western blotting after siRNA depletion (Extended Data Fig. 6f,g).<br>The GFP ab290 antibody has been extensively used to perform ChIP-seq, such as in Gal et al. 2021 Cell Reports, and is further validated as per the manufacturer's website: https://www.abcam.com/gfp-antibody-ab290.html<br>The RPB1 14958S antibody is validated as per the manufacturer's website: https://www.cellsignal.com/products/primary-antibodies/rpb1-ntd-d8l4y-rabbit-mab/14958?N=0+102236+4294956287&Nrpp=200&No=4600&fromPage=plp<br>The GAPDH 2118S antibody is validated as per the manufacturer's website: https://www.cellsignal.com/products/primary-antibodies/gapdh-14c10-rabbit-mab/2118<br>The His6 ab18184 antibody is validated as per the manufacturer's website: https://www.abcam.com/6x-his-tag-antibody-hish8-ab18184.html<br>The H3 ab1791 antibody is validated as per the manufacturer's website: https://www.abcam.com/histone-h3-antibody-nuclear-marker-and-chip-grade-ab1791.html

Eukaryotic cell lines

Policy information about cell lines and Sex and Gender in Research

Cell line source(s)	U-2 OS cells were obtained from ATCC. HEK293 cells were obtained from Kristian Helin's lab. U-2 OS Flp-In cells were obtained from Karmella Haynes' lab.
Authentication	U-2 OS Flp-In cells over-expressing different versions of GFP-tagged SPT2 were validated by western blotting. Confirmation of the knock-out of the SPTY2D1 gene was performed by western blot and by sequencing.
Mycoplasma contamination	All human cell lines used in this study were routinely tested negative for mycoplasma.
Commonly misidentified lines<br>(See ICLAC register)	No commonly misidentified cells were used in this study.

Animals and other research organisms

Policy information about studies involving animals; ARRIVE guidelines recommended for reporting animal research, and Sex and Gender in Research

Laboratory animals	Caenorhabditis elegans nematodes. In our Methods section, we indicate the 'age' of the C. elegans worms by stating the larval stage at which the worms are used; for adult worms, we state how many days post-larval stage the worms were used.
Wild animals	No wild animals were involved in the study.
Reporting on sex	The C. elegans nematodes used in this study are hermaphrodites.<br>U-2 OS human cells are derived from a female donor.
Field-collected samples	No samples were collected from the field.
Ethics oversight	No ethical approval was required for working with C. elegans animals.

Note that full information on the approval of the study protocol must also be provided in the manuscript.

ChIP-seq

Data deposition

☒ Confirm that both raw and final processed data have been deposited in a public database such as GEO.

☒ Confirm that you have deposited or provided access to graph files (e.g. BED files) for the called peaks.

Data access links
May remain private before publication.

https://www.ncbi.nlm.nih.gov/geo/query/acc.cgi?acc=GSE224802

Files in database submission

GSE243274 Investigating how the SPT2 histone chaperone affects chromatin structure in human osteosarcoma cells.
GSM7782685 WT, replicate 1
GSM7782686 WT, replicate 2
GSM7782687 WT, replicate 3
GSM7782688 SPT2 KO-3, replicate 1
GSM7782689 SPT2 KO-3, replicate 2
GSM7782690 SPT2 KO-3, replicate 3
GSM7782691 SPT2 KO-4, replicate 1
GSM7782692 SPT2 KO-4, replicate 2
GSM7782693 SPT2 KO-4, replicate 3
GSM7782694 SPT2 KO-6, replicate 2
GSM7782695 SPT2 KO-6, replicate 3

GSE224799 Investigating how the SPT-2 histone chaperone affects chromatin structure in C. elegans.
GSM7032552 WT, replicate 1
GSM7032553 WT, replicate 2
GSM7032554 spt-2 KO-A, replicate 1
GSM7032555 spt-2 KO-A, replicate 2
GSM7032556 spt-2 M627A, replicate 1
GSM7032557 spt-2 M627A, replicate 2

GSE224800 Identification of GFP-SPT-2 binding sites in the Caenorhabditis elegans genome
GSM7032558 gfp::spt-2 C. elegans, GFP, replicate 1
GSM7032559 gfp::spt-2 C. elegans, GFP, replicate 2
GSM7032560 WT N2 C. elegans, GFP, replicate 1
GSM7032561 WT N2 C. elegans, GFP, replicate 2
GSM7032562 WT N2 C. elegans, input
GSM7032563 GFP-SPT-2 C. elegans, input

GSE224801 Differential gene expression in spt-2 mutant worms.
GSM7032564 WT, 20C, replicate 1
GSM7032565 WT, 20C, replicate 3
GSM7032566 spt-2 KO A, 20C, replicate 1
GSM7032567 spt-2 KO A, 20C, replicate 3
GSM7032568 spt-2 KO B, 20C, replicate 1
GSM7032569 spt-2 KO B, 20C, replicate 2
GSM7032570 spt-2 KO C, 20C, replicate 3
GSM7032571 WT, 20C, replicate 4
GSM7032572 WT, 20C, replicate 5
GSM7032573 WT, 20C, replicate 6
GSM7032574 spt-2 M627A, 20C, replicate 1
GSM7032575 spt-2 M627A, 20C, replicate 2

Genome browser session
(e.g. UCSC)

BigWig files have been provided in the GEO database.

Methodology

Replicates

Two independent replicates of the ChIP-seq experiment were performed, with worms from the two replicates collected independently on different days.

Sequencing depth

- GFP IP (gfp::spt2) rep 1 32,169,120 pairs
- GFP IP (gfp::spt2) rep 2 29,237,604 pairs
- GFP IP (WT) rep 1 24,313,284 pairs
- GFP IP (WT) rep 2 28,602,884 pairs
- input gfp::spt-2 29,410,305 pairs
- input WT 28,175,667 pairs

Antibodies

GFP antibody: ab290

Peak calling parameters

CeSPT-2 ChIP-seq peaks were called using MACS2 (settings: --SPMR --gsize ce --keep-dup all --nomodel --broad) using the GFP ChIP-seq samples from wild type animals as controls. The final SPT-2 peak set was defined by the intersection of the broad peaks called on

each replicate.
A gene was considered an CeSPT-2 target if more than 50% of the length of its longest annotated transcript was covered by a CeSPT-2 peak. The average CeSPT-2 coverage over CeSPT-2 targets was calculated using coverageBed from the BEDTools suite77 (v.2.30.0). Coverage plots over gene models were produced using the DeepTools suite78 (version 3.5.1).

Data quality

ChIP-seq reads were preprocessed using trim-galore (version 0.6.7) and mapped on the C. elegans genome (wormbase release WS285) using bwa-mem (version 0.7.17)70. Reads with mapping quality (MAPQ) higher than 10 were extracted using Samtools71. ChIP-seq peaks were called using MACS2 (settings: --SPMR --gsize ce --keep-dup all --nomodel --broad).

Software

Trim-galore (version 0.6.7); bwa-mem (version 0.7.17); BEDTools suite75 (v.2.30.0); DeepTools suite76 (version 3.5.1).
